# Mathematical modelling to centre low tidal volumes following acute lung injury: A study with biologically variable ventilation

**DOI:** 10.1186/1465-9921-6-64

**Published:** 2005-06-28

**Authors:** M Ruth Graham, Craig J Haberman, John F Brewster, Linda G Girling, Bruce M McManus, W Alan C Mutch

**Affiliations:** 1 Department of Anesthesia, University of Manitoba, Winnipeg, Manitoba, Canada; 2 Institute of Industrial Mathematical Sciences, University of Manitoba, Winnipeg, Manitoba, Canada; 3 Department of Pathology and Laboratory Medicine, James Hogg iCAPTURE Centre for Cardiovascular and Pulmonary Research, University of British Columbia, Vancouver, British Columbia, Canada

## Abstract

**Background:**

With biologically variable ventilation [BVV – using a computer-controller to add breath-to-breath variability to respiratory frequency (*f*) and tidal volume (V_T_)] gas exchange and respiratory mechanics were compared using the ARDSNet low V_T _algorithm (Control) versus an approach using mathematical modelling to individually optimise V_T _at the point of maximal compliance change on the convex portion of the inspiratory pressure-volume (P-V) curve (Experimental).

**Methods:**

Pigs (n = 22) received pentothal/midazolam anaesthesia, oleic acid lung injury, then inspiratory P-V curve fitting to the four-parameter logistic Venegas equation *F*(*P*) = *a *+ *b*[1 + *e*^-(*P*-*c*)/*d*^]^-1 ^where: *a *= volume at lower asymptote, *b *= the vital capacity or the total change in volume between the lower and upper asymptotes, *c *= pressure at the inflection point and *d *= index related to linear compliance. Both groups received BVV with gas exchange and respiratory mechanics measured hourly for 5 hrs. Postmortem bronchoalveolar fluid was analysed for interleukin-8 (IL-8).

**Results:**

All P-V curves fit the Venegas equation (R^2 ^> 0.995). Control V_T _averaged 7.4 ± 0.4 mL/kg as compared to Experimental 9.5 ± 1.6 mL/kg (range 6.6 – 10.8 mL/kg; p < 0.05). Variable V_T_s were within the convex portion of the P-V curve. In such circumstances, Jensen's inequality states "if *F*(*P*) is a convex function defined on an interval (*r*, *s*), and if *P *is a random variable taking values in (*r*, *s*), then the average or expected value (*E*) of *F*(*P*); *E*(*F*(*P*)) > *F*(*E*(*P*))." In both groups the inequality applied, since *F*(*P*) defines volume in the Venegas equation and (*P*) pressure and the range of V_T_s varied within the convex interval for individual P-V curves. Over 5 hrs, there were no significant differences between groups in minute ventilation, airway pressure, blood gases, haemodynamics, respiratory compliance or IL-8 concentrations.

**Conclusion:**

No difference between groups is a consequence of BVV occurring on the convex interval for individualised Venegas P-V curves in all experiments irrespective of group. Jensen's inequality provides theoretical proof of why a variable ventilatory approach is advantageous under these circumstances. When using BVV, with V_T _centred by Venegas P-V curve analysis at the point of maximal compliance change, some leeway in low V_T _settings beyond ARDSNet protocols may be possible in acute lung injury. This study also shows that in this model, the standard ARDSNet algorithm assures ventilation occurs on the convex portion of the P-V curve.

## Background

Mathematical modelling has contributed to our understanding of lung mechanics and helped direct therapy in patients with acute respiratory distress syndrome (ARDS). Hickling [1,2] generated sigmoidal pressure-volume (P-V) curves based on a model where airway opening could occur over the entire inflation limb. Venegas and colleagues [3,4] devised a four-parameter logistic model to fit P-V inflation curves in patients with ARDS. In most instances, the Venegas equation fits static P-V data with great precision. Such modelling indicates that ventilation is limited to the convex portion of the static inflation curve when the low tidal volume (V_T_) ARDSNet algorithm is utilised [5].

We have recently shown mathematically that if ventilation is occurring on the convex portion of the P-V curve, there is an advantage to adding noise to the end-inspiratory pressure signal [6,7]. Using a newer mode of mechanical ventilation – termed biologically variable ventilation (BVV) – noise is added to the end-inspiratory pressure signal [8]. As configured this noise can be shown to have fractal or 1/f characteristics [9]. This computer-controlled ventilator simulates breath-to-breath variation in respiratory frequency (*f*) and V_T _that characterises normal spontaneous ventilation. The added noise results in greater mean V_T _over time at the same mean driving pressure. This, perhaps counter-intuitive finding, can be deduced by applying Jensen's inequality – a simple probabilistic proof [7,10]. Jensen's inequality states that the average or expected value of a convex function over a random variable is greater than the value of that function at the average of the random variable. In mathematical terms in the notation of the Venegas equation, "if *F*(*P*) = *V *is a convex function defined on an interval (*r*, *s*), and if pressure (*P*) is a random variable taking values in (*r*, *s*), then the expected value (*E*) at *F*(*P*); *E*(*F*(*P*)) > *F *at the expected value of *P*; *F*(*E*(*P*))." Such conditions are met with BVV since noisy ventilation provides a series of individualised observations of pressure (*P*), that are transformed to volume *F*(*P*) as determined by Venegas curve fitting.

Jensen's inequality, thus, provides us with an important tool to determine if noise will be beneficial or not. Indirectly it also indicates where the noise will be most beneficial – when ventilation is centred at the point where the convexity is most pronounced – the point where the second derivative of the convex interval of the function is maximised. For the Venegas equation this occurs at the point of maximal compliance change: when *P *= *c *- 1.317*d *or when *V *= *a *+ 0.211*b *[3,6].

Based on the above information, we designed an experiment to compare the presumed "mathematically optimised" point about which to centre noise (Experimental) to an approach using the ARDSNet algorithm (Control). We presumed that the ARDSNet algorithm would also result in ventilation on the convex portion of the P-V curve, but advanced the hypothesis that by mathematical modelling individual P-V curves, we could find an optimised strategy for BVV that would result in discernable improvements over an approach using the ARDSNet algorithm alone with BVV. A porcine model of lung injury with oleic acid was studied. We compared gas exchange, respiratory mechanics and a single marker of inflammation over 5 hrs for the two approaches.

## Methods

### Experimental preparation

Twenty-eight pigs were studied following the Canadian Council on Animal Care Guidelines. The experimental preparation has been described previously [11]. Briefly, animals were ventilated initially with an Esprit^® ^ventilator (Respironics Inc., Carlsbad, CA) using V_T _= 12 mL/kg, *f *= 20 bpm, F_I_O_2 _0.5 and PEEP 4 cm H_2_O during surgical placement of monitoring cannulae. Anaesthesia was maintained with an intravenous loading dose and continuous infusion of sodium thiopental/midazolam at 16/0.1 mg/kg/hr and paralysis with doxacurium infusion (1.5 – 2 mg/kg/hr).

### Oleic Acid Lung Injury

Baseline measurements were obtained and an infusion of oleic acid (BDH, Toronto, ON) started at 0.2 mL/kg/hr through a catheter, placed in the inferior vena cava, above the level of the diaphragm. The oleic acid infusion was continued until PaO_2 _decreased to <80 mm Hg for two consecutive measurements, 5 min apart (20 – 45 min of infusion), and the volume noted. An additional 4 cm H_2_O PEEP was then added (to a total of 8 cm H_2_O) and arterial blood gases were obtained at 10 and 15 min. The criterion for study inclusion was a PaO_2 _> 80 mmHg and <200 mmHg on 8 cm H_2_O of PEEP. This was not considered to represent steady-state but was used as an index of adequate lung injury (PaO_2 _< 80 mm Hg) as well as evidence for lung recruitability (80 < PaO_2 _< 200 mm Hg). A continuous infusion of dopamine (5 – 10 μg/kg/min) was started with oleic acid infusion to maintain mean arterial pressure > 50 mmHg.

### Static Pressure-Volume Curves

Pressure-volume curves were generated for each animal after established lung injury. F_I_O_2 _was increased to 1.0, PEEP decreased to 0 cm H_2_O, and *f *was decreased to 10 bpm with an inspiratory hold of 2 sec, at a square-wave flow rate of 30 L/min. A sequence of V_T_s from 50 to 1200 mL was delivered and plateau pressure measured 1 sec after end inspiration. Preliminary trials yielded similar curves with V_T _delivered in either random or ascending sequences, so the latter was used. The resulting P-V curve was analysed using a non-linear regression curve-fitting program (NCSS 97) that performs a series of iterations. We used the four-parameter logistic equation derived by Venegas et al. [3] to curve fit:

*F*(*P*) = *a *+ *b*[1 + *e*^-(*P*-*c*)/*d*^]^-1^

where: *a *= volume at the lower asymptote, *b *= the vital capacity or the total change in volume between the lower and upper asymptotes, *c *= pressure at the true inflection point and *d *= an index of the linear compliance for the curve. The maximal rate of change in compliance is the point where the second derivative is maximal: found at the point *P *= *c *- 1.317*d *or *V *= *a *+ 0.211*b*.

### Ventilation Protocol

**Figure 1:**
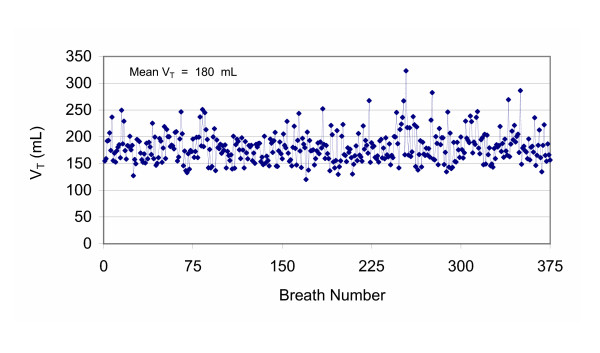
**Delivery of Variable Tidal Volume**. The complete data set of delivered tidal volume (V_T_) using BVV in one animal. There were 376 breaths in the file. Mean V_T _was set at 180 mL in this example.

The animals were then randomised to BVV centred at V_T _of 7 mL/kg, (Control) or at the V_T _corresponding to the maximal rate of change in compliance from the P-V curve (Experimental) for 5 hrs. We used the computer-controller and software to generate the variable ventilatory pattern as previously described [11]. A representative variability file is shown in Figure 1. F_I_O_2 _was set at 0.5 with PEEP 8 cm H_2_O. Respiratory frequency was initially set at 25 bpm. We followed the ARDSNet algorithm for pH control – the base V_T _of 7 mL/kg in the Control group is greater than the 6 mL/kg seen in human studies due, in part, to dead space associated with in-line breathing circuit measurement devices. If pH fell below 7.2, *f *was incrementally increased by 5 bpm up to a maximum of 35 bpm. If respiratory acidosis persisted, V_T _could be adjusted in increments of 0.5 mL/kg to a maximum of 8 mL/kg. Haemodynamics, airway pressures, arterial and venous blood gases and static compliance were determined at baseline, after oleic acid, after generation of the P-V curve and then hourly for 5 hrs.

### Bronchoalveolar Fluid Cytokines and Wet/Dry Weight Ratios

Bronchoalveolar fluid aspirates were obtained immediately post-mortem. These samples were frozen and kept at -80°C until analysis. Analyses were made in duplicate to determine the concentrations of IL-8 by sandwich ELISA. A species-specific assay was used (IL-8, Medicorp KSC0082, detection limit 10 pg/mL). ELISA plates were incubated at 4°C overnight with 50 μL per well with 1 mg/mL of anti-IL-8. Plates were washed 4 times and nonspecific binding was blocked with 200 μL of phosphate-buffered saline (PBS) with 2% bovine serum albumin (BSA) per well for 90 min. Diluted cell-free supernatants (50 μL) were added and incubated for 3 hr. A volume of 50 μL (1 mg/mL) of biotinylated antibody was added and incubated for 60 min. Subsequently, avidin peroxidase conjugate was added (Bio-Rad Laboratories) followed by chromogen substrate (ortho-phenylenediamine [OPD], Dako). Plates were read at 490 nm using an ELISA reader (Rainbow Reader, SLT Lab Instruments). The analysis of aspirates was done in a blinded fashion at the James Hogg iCAPTURE Centre for Cardiovascular and Pulmonary Research, University of British Columbia.

### Statistical Analysis

Data were analysed by repeated measures analysis of variance (ANOVA) as previously described. The group × time interactions were considered significant when p < 0.05. Least squares means test matrices were generated for post-hoc comparisons and Bonferroni's correction applied for multiple comparisons within groups. Single between group comparisons were by unpaired t-test; p < 0.05 considered significant.

## Results

Four pigs died after generation of the P-V curve or within one hr of initiation of mechanical ventilation due to profound hypoxaemia and were excluded from analysis. Two pigs were excluded prior to randomisation for failure to meet blood gas criteria leaving 22 animals that completed the protocol, 11 in each group. There were no differences in body weight, volume of oleic acid infused, or dopamine dose administered between groups.

### Venegas Equation Curve Fitting

**Figure 2:**
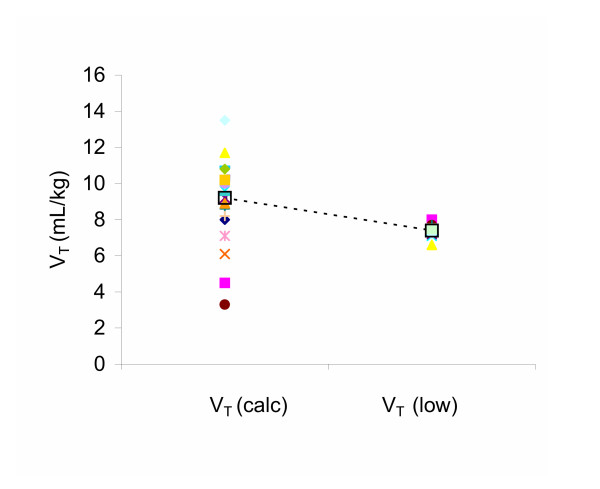
**Mathematically Calculated versus Algorithm Low Tidal Volume**. V_T _calculated from the point of maximal compliance change (*c *- 1.317*d*) on the P-V curve for all animals (left hand points) as compared to Control group (ARDSNet algorithm) low V_T _(right hand points). Mean V_T _of each group given by large open square, connected by the dotted line.

All curves fit the Venegas equation with R^2 ^> 0.995. The derived Venegas parameters for all animals are shown in Table 1. In the Control group, average V_T _was 7.4 ± 0.4 mL/kg. This was higher than the 7 mL/kg target due to increased V_T _in 6 of 11 animals to control pH following oleic acid injury as per the ARDSNet algorithm. In the Experimental group average V_T_, optimised to the point of maximal compliance change, was 9.5 ± 1.6 mL/kg, significantly higher than in the Control group (p < 0.05). In the Experimental group, the average 95% margin of error for the target V_T _was 1.06 mL/kg. Of the 11 animals in this group, 10 had V_T _values higher than the ARDSNet protocol, and 9 of the associated 95% confidence intervals did not contain 7.0 mL/kg. One animal had a target V_T _below 7.0 mL/kg, although the associated confidence interval contained 7.0 mL/kg. Combining all 22 animals as one group, values for V_T _calculated from the Venegas equation at *P *= *c *- 1.317*d *or *V *= *a *+ 0.211*b *varied from a low of 3.3 mL/kg to a high of 13.5 mL/kg (Figure 2). This targeted V_T _was greater than 7 mL/kg in 18/22 animals. In the Experimental group alone, the range of individualized V_T_s was 6.6 -10.8 mL/kg. The corresponding Paw at the point *c *- 1.317*d *for each animal is also shown in Table 1. The range of Paw at this point showed substantial inter-animal variability ranging from 8.4 to 20.8 cm H_2_O, with a mean of 15.9 ± 3.4 cm H_2_O overall.

**Table 1:** Venegas Parameters

CONTROL GROUP								EXPERIMENTAL GROUP							
	Venegas Parameters								Venegas Parameters						
	*a*	*b*	*c*	*d*	V at c-1.317d (mL/kg)	V_T _delivered (mL/kg)	P at c-1.317d (cmH_2_O)		*a*	*b*	*c*	*d*	V at c-1.317d (mLl/kg)	V_T _delivered (mL/kg)	P at c-1.317d (cmH_2_O)

1	-38.6	1165	22.9	6.8	8	7.5	14	1	-223.3	2332.3	35.3	15.8	10.2	10.1	14.5
2	-213.6	1562.9	28.5	15.3	4.5	8	8.4	2	-97.9	1652	30.7	11.9	10.5	9.7	15
3	-41.8	1472.3	26.2	9	11.7	6.6	14.5	3	-151.8	1461.8	25.4	11.3	7.1	7.1	10.5
4	-98.7	1600	25.8	7.9	9.6	7.1	15.4	4	-116.5	1645.5	26.4	9.8	10	10.9	13.5
5	-25.1	1292.2	29.2	7.9	9.2	7.2	18.7	5	-46.5	1303.4	24.1	6.4	8.2	7.9	15.7
6	-168	1152.3	27.9	12.1	3.3	7.7	12.1	6	-72.1	1400.4	31.5	9.4	10.2	11.3	19.1
7	-54	1232.8	31.9	8.4	8.6	7.7	20.8	7	-0.38	1159.6	31.4	8.1	9.4	9.8	20.7
8	-223.9	2295.2	34.5	15.4	10.4	7.5	14.3	8	-51	1573.6	25.8	8.7	10.8	11	14.3
9	10.8	1087.4	27.4	6.2	10.9	7.2	19.2	9	-23.3	1319.6	24.5	6.2	10.2	10.5	16.3
10	-64.5	1837.8	34.4	10.9	13.5	7.2	20	10	-16.6	1163.4	29.5	9.4	8.9	9.4	17.1
11	-46.7	1563.2	25.8	8.7	10.1	7.5	14.4	11	-62.2	1015.2	31.1	7.8	6.1	6.6	20.9

MEAN	-87.6	1478.3	28.6	9.9	9.1	7.4	15.6	MEAN	-78.3	1457.0	28.7	9.5	9.2	9.5	16.1
SD	79.0	357.5	3.7	3.2	3.0	0.4	3.7	SD	65.7	356.2	3.6	2.7	1.5	1.6	3.2

**Figure 3:**
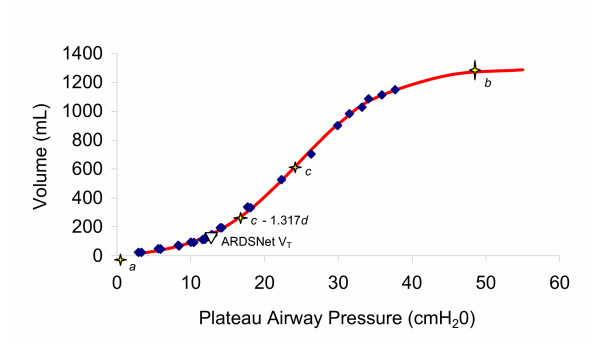
**Representative Pressure-Volume Curve Fit to the Venegas Equation**. Representative P-V curve generated at zero end expiratory pressure in a single animal in the Experimental group. Dots are individual data points. The line represents the Venegas equation derived P-V curve. The Venegas parameters *a*, *c *and *b *are labelled, as well as the volume at the point of maximal compliance change (*P *= *c *- 1.317*d*) and the volume equivalent to 7 mL/kg in this animal. See text for further explanation.

A representative P-V curve is shown in Figure 3, corresponding to animal 9 in the Experimental group. For this animal, the estimated parameters in the Venegas model were given by *a *= -23.3 mL, *b *= 1319.6 mL, *c *= 24.5 cm H_2_O, and *d *= 6.2 cm H_2_O. The data were collected over a wide range of pressures and volumes, and the model provided a good fit to the data (R^2 ^= 0.996). The mean volume at the point of maximal compliance change was estimated to be V_T _= (*a *+ 0.211*b*)/w = 10.2 mL/kg, (weight = 25 kg). The associated 95% margin of error was 0.54 mL/kg, derived from the estimated variance-covariance matrix for the calculated parameters. Thus the 95% confidence interval for this volume was 9.7 to 10.8 mL/kg, which, in this example, does not encompass the 7.0 mL/kg V_T _of the Control group.

### Variability Frequency Distribution

**Figure 4:**
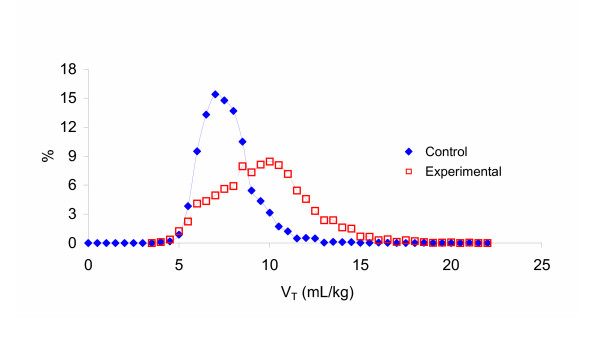
**Frequency Distribution Curves for Tidal Volume for the Two Groups**. Frequency distribution curves of V_T _for each group calculated in bins of 0.5 mL/kg. The V_T _bins are represented on the x-axis and the percentage of all V_T_s from each group in each bin is represented on the y-axis. Control = solid diamonds. Experimental = open squares.

The variability file for BVV introduced a coefficient of variation in V_T _of 15%. This translated into overall fluctuations in V_T _between 4.1 – 11.1 mL/kg in the Control group and 3.4 – 16.8 mL/kg in the Experimental group. Figure 4 shows the frequency distribution of V_T_s for each group calculated in bins of 0.5 mL/kg. A broader distribution was present in the Experimental group due to the greater range of initial centring V_T_s (6.6 – 11.3 mL/kg vs. 6.6 – 8 mL/kg) but it is evident that the addition of a variable ventilation pattern to both groups results in substantial V_T _overlap between groups.

### Minute Ventilation and Airway Pressures

**Figure 5:**
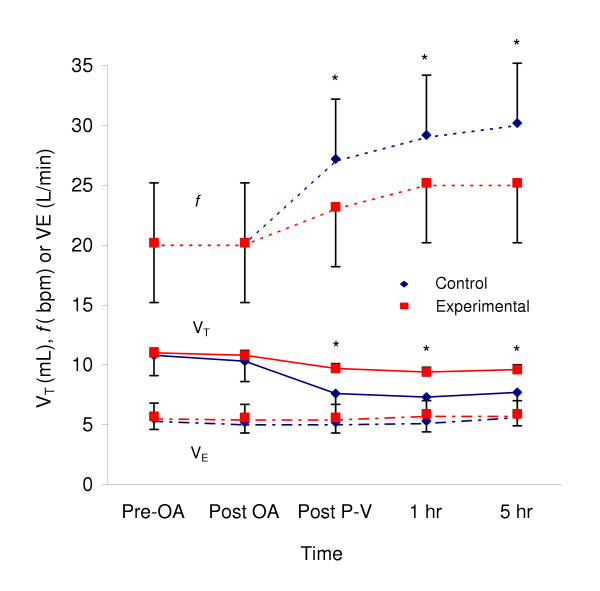
**Ventilation Parameter for the Control and Experimental Groups**. Mean values of V_T_, *f*, and minute ventilation (V_E_) for Control (diamond symbol) and Experimental groups (square symbol) at each time period. Bars represent standard deviation. * p < 0.05 between groups at specified time periods.

**Figure 6:**
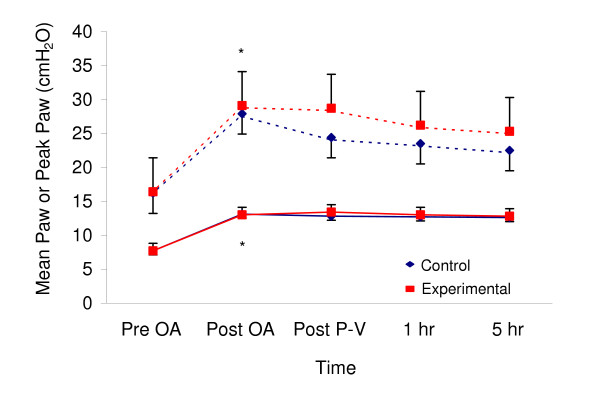
**Mean Peak and Mean Airway Pressure for the Two Groups**. Mean values for peak and mean Paw for Control (diamond) and Experimental (square) groups at each time period. Bars represent standard deviation. *p < 0.05 following oleic acid.

Equivalent minute ventilation was maintained in both groups at all times. This required a statistically significant increase in *f *to 30 ± 5 bpm in the Control group compared to 25 ± 6 bpm (p < 0.05) in the Experimental group from hr 1 to hr 5 (Figure 5). Peak and mean Paw increased to a similar extent after oleic acid infusion in both groups. Peak Paw was modestly, but not significantly, higher in the Experimental group compared to Control (25.0 and 25.9 cm H_2_O at 1 and 5 hrs respectively vs. 23.2 and 22.2 cm H_2_O at analogous time periods in Control) (Figure 6). Mean Paw was approximately 7.5 cm H_2_O at baseline and increased to 12.5 cm H_2_O after oleic acid, not different between groups at any time-period.

In each animal, plateau pressure was determined by clamping the expiratory line for 12 – 18 individual breaths over the five hr period. Using this technique, plateau pressures were well within the "safe" range of <30 cm H_2_O in both groups: 22.3 ± 3.7 vs. 24.5 ± 4.7 cm H_2_O in the Control and Experimental groups respectively. Even mean peak airway pressures were below this theoretical upper limit (Figure 6). As well, plateau pressures were below the calculated value for the true inflection point *c *(mean value 28.6 ± 3.7 cm H_2_O and 28.7 ± 3.6 cm H_2_O in the Control and Experimental groups respectively), except for a single measurement in each 376-breath cycle of the program file (see Figure 1 for demonstration of the largest V_T_).

### Respiratory Gases and Mechanics

Data are shown in Table 2. Similar changes in arterial blood gases occurred with oleic acid administration in each group. PaO_2 _decreased from approximately 250 mmHg to approximately 100 mmHg with oleic acid infusion in both groups, then it increased over 5 hrs to a mean of greater than 150 mmHg with no differences between groups. Following oleic acid injury, the PaCO_2 _increased significantly in both groups and remained elevated with no between group differences. Mixed venous O_2 _(PvO_2_) decreased with oleic acid injury and remained depressed over the duration of the experiment in both groups. Arterial pH fell from 7.4 at control to 7.3 after P-V curve determination in both groups, associated with the respiratory acidosis. After 1 hr of ventilation, with adjustments in *f *and V_T_, PaCO_2 _showed evidence of normalisation and pH improved in both groups for the duration of the study. Respiratory system compliance (Crs) decreased significantly with oleic acid administration and decreased further after P-V curve analysis, with no difference between groups and no group × time interaction.

**Table 2:** Respiratory Gases and Mechanics

	Pre OA	Post OA	Post PV Curve	1 hr	5 hr
PaO_2_					
Control Group	265 ± 23 *	101 ± 15	113 ± 9	147 ± 46 *	176 ± 36 *
Experimental Group	267 ± 19 *	89 ± 16	100 ± 45	139 ± 59 *	163 ± 62 *
					
PaCO_2_					
Control Group	38 ± 4 *	48 ± 5	56 ± 9	57 ± 7	51 ± 5
Experimental Group	38 ± 2 *	48 ± 7	53 ± 10	54 ± 21	49 ± 12
					
PvO_2_					
Control Group	43 ± 4 *	35 ± 3	38 ± 2	37 ± 5	35 ± 4
Experimental Group	48 ± 5 *	38 ± 5	37 ± 5	39 ± 7	36 ± 6
					
pHa					
Control Group	7.45 ± 0.04 *	7.34 ± 0.04	7.30 ± 0.07	7.29 ± 0.06	7.35 ± 0.04
Experimental Group	7.45 ± 0.03 *	7.33 ± 0.04	7.30 ± 0.08	7.30 ± 0.08	7.35 ± 0.07
					
Crs					
Control Group	1.15 ± 0.17 *	0.66 ± 0.12	0.58 ± 0.12	0.55 ± 0.13	0.61 ± 0.11
Experimental Group	1.11 ± 0.17 *	0.70 ± 0.22	0.58 ± 0.17	0.60 ± 0.16	0.68 ± 0.18

PaO_2 _= Arterial Partial Pressure O_2 _(mmHg)

PaCO_2 _= Arterial Partial Pressure CO_2 _(mmHg)

PvO_2 _= Mixed Venous Partial Pressure O_2 _(mmHg)

pHa = Arterial pH

Crs = Compliance L/cmH_2_O

* p < .05 Within Groups vs Post PV Curve

Control Group n = 11 Mean ± SD

Experimental Group n = 11 Mean ± SD

### Haemodynamic Data

Results are shown in Table 3. Mean arterial pressure (MAP) did not differ between groups at any time period. With P-V curve generation, mean pulmonary artery pressure (MPAP) increased over baseline but returned towards baseline values at end experiment in both groups. Pulmonary artery occlusion pressure (PAOP) did not differ between groups at any time-period. Pulmonary vascular resistance (PVR) essentially tripled with oleic acid injury and following generation of the P-V curve with some return towards normal over time in both groups. Cardiac output (CO) decreased significantly with oleic acid injury and remained depressed in both groups for the duration of the experiment.

**Table 3:** Haemodynamic Data

	Pre OA	Post OA	Post PV Curve	1 hr	5 hr
MAP					
Control Group	108 ± 17	101 ± 11	111 ± 19	114 ± 21	99 ± 29
Experimental Group	119 ± 15	108 ± 25	122 ± 27	122 ± 25	104 ± 24
					
MPAP					
Control Group	24 ± 9 *	35 ± 6	36 ± 8	32 ± 5	28 ± 3 *
Experimental Group	23 ± 5 *	35 ± 6	34 ± 7	33 ± 7	29 ± 7 *
					
PAOP					
Control Group	9.3 ± 1.5	10.5 ± 1.9	9.9 ± 1.1	9.9 ± 1.3	9.6 ± 1.5
Experimental Group	10.0 ± 1.7	10.6 ± 1.4	11.0 ± 1.5	10.8 ± 1.5	11.1 ± 1.5
					
PVR					
Control Group	4.3 ± 2.6 *	11.1 ± 4.0	12.4 ± 6.0	12.3 ± 4.6	8.5 ± 2.8 *
Experimental Group	3.2 ± 1.5 *	8.6 ± 2.0	8.8 ± 3.0	9.8 ± 3.1	7.6 ± 3.0 *
					
CO					
Control Group	3.5 ± 0.7 *	2.3 ± 0.5	2.3 ± 0.6	2.0 ± 0.5	2.2 ± 0.6
Experimental Group	4.2 ± 0.8 *	2.9 ± 0.5	2.8 ± 0.6	2.5 ± 0.5	2.4 ± 0.5

MAP = Mean Arterial Pressure (mmHg)

MPAP = Mean Pulmonary Arterial Pressure (mmHg)

PAOP = Pulmonary Artery Occlusion Pressure (mmHg)

PVR = Pulmonary Vascular Resistance (mmHg·L·min^-1^)

CO = Cardiac Output (L/min)

* p < .05 Within Groups vs Post PV Curve

Control Group n = 11 Mean ± SD

Experimental Group n = 11 Mean ± S.D.

### Bronchoalveolar Fluid Inflammatory Mediators

The average concentration of IL-8 in tracheal aspirate was 5510 ± 2540 pg/mL in the Control group versus 6500 ± 2440 pg/mL in the Experimental group, not different between groups by unpaired t-test (t statistic = -0.866, p = 0.397).

## Discussion

With BVV, when "mathematically optimising" V_T _to the point of maximal compliance change on the convex portion of the P-V curve, no statistical improvement over the ARDSNet algorithm for V_T _selection was seen for oxygenation, respiratory mechanics or inflammatory cytokines in this animal model of ARDS. With either approach, acceptable gas exchange was maintained over 5 hrs and no significant differences in ventilating pressures, respiratory mechanics, dead space, shunt fraction or IL-8 cytokine levels were seen. We did not include a group with low V_T _in control mode as a previous study demonstrated significant advantages with BVV, both using the ARDSNet protocol [12]. Four mechanisms have been invoked to account for these advantages: i) stochastic resonance (noise enhancement of an input signal) [13], ii) Jensen's inequality [6], iii) increased surfactant [14] and iv) enhanced respiratory sinus arrhythmia[9]. The goal of the present study was therefore, not to examine BVV mechanistically, but to determine if the benefits seen previously with BVV could be optimised based on fitting the Venegas equation to individual P-V curves and then determining the ideal point on the convex portion of the curve about which to ventilate.

Using the "mathematically optimised" approach yielded mean V_T_s that were higher than the ARDSNet algorithm (see Figure 2). The majority of calculated V_T_s were between 8 and 10 ml/kg, and both Control and Experimental V_T_s were within the convexity of their individualised P-V curves. By ventilating with BVV in both groups, which introduced a coefficient of variation in V_T _of 15%, a substantial overlap of delivered V_T _was seen – apparent in the frequency distributions of V_T _(see Figure 4). We could not know *a priori *what V_T _calculated at the point *P *= *c *- 1.317*d *or *V *= *a *+ 0.211*b *would be relative to Control V_T _but their proximity, coupled with the extensive overlap due to the addition of BVV, contributed to the lack of discernable difference between groups.

The average "mathematically optimised" V_T _is marginally higher than that currently recommended by proponents of low V_T _ventilation. However, this V_T _is within the range chosen by most intensive care units managing ARDS patients worldwide [15] as well as the V_T _selected for the control arm of the three clinical trials showing no benefit from V_T _reduction (Control V_T _= 10.8, 10.3 and 10.2 mL/kg) [16-18]. The highest derived V_T _from Venegas curve fitting (13.5 ml/kg) approached what some clinicians may consider unsafe in ARDS patients using conventional ventilation [19]. However, use of higher V_T_s in combination with BVV did not significantly increase airway pressures or IL-8 concentrations compared to Control and minute ventilation could be maintained at lower *f*. Lower *f *may ameliorate gas trapping that has been demonstrated in ARDS patients ventilated at *f *greater than 30 bpm [20]. Although unable to demonstrate an advantage by "mathematically optimising" V_T _in the Experimental group, knowledge of the point of maximal compliance in combination with BVV may provide greater flexibility in choosing ventilator settings in individual patients, permitting a higher V_T_/lower *f *combination while maintaining acceptable airway pressures.

Noisy ventilation can demonstrate Jensen's inequality in two ways. Experiments have shown that with BVV, expected mean V_T _is greater at the same mean Paw [21] or, conversely, that expected Paw is lower at the same mean V_T _[12]. That is, the noise can be introduced in either pressure or volume. In the latter case, the concavity of the inverse function



is being exploited in the region of low volumes. Thus, a lower expected plateau pressure for a given mean V_T _with BVV is also a consequence of Jensen's inequality.

Current recommendations to limit ventilator induced lung injury (VILI) include a combination of low V_T _and adequate PEEP to maintain an open lung with plateau pressures below 30 cm H_2_O. The plateau pressures over the course of this experiment, determined by clamping the expiratory line in 12 – 18 individual breaths per experiment, were well within the "safe" range in both groups: 22.3 ± 3.7 vs. 24.5 ± 4.7 cm H_2_O in the Control and Experimental groups respectively. Plateau pressures were also below the calculated value for the true inflection point *c *on the Venegas curve (mean value 28.6 ± 3.7 cm H_2_O and 28.7 ± 3.6 cm H_2_O in the Control and Experimental groups respectively). Even mean peak airway pressures were below this theoretical upper limit (Figure 6). In each experiment, a single breath exceeded point *c *(the upper bound for the convexity of the P-V curve). The average V_T _of this single largest breath delivered once in the file of 376 breaths (Figure 1) for the Experimental group was 426 ± 79 mL associated with a peak Paw of 42 ± 9 cm H_2_O at PEEP 8 cm H_2_O.

Brower et al. [22] recently re-analysed the ARDSNet data by quartiles for plateau pressure and found that V_T _reduction was associated with reduced mortality in all patients, including those with plateau pressures less than 32 cm H_2_O. These authors contend that there is no "safe" level of plateau pressure in acute lung injury and the lowest V_T _and plateau pressure compatible with acceptable gas exchange should be a goal. If this is borne out, then application of a variable ventilator pattern, which has consistently resulted in improved gas exchange and respiratory mechanics at airway pressures that were equivalent to or lower than those obtained during conventional ventilation, in combination with the lowest V_T_, may be beneficial.

The risk of strict adherence to low V_T _with conventional ventilation is alveolar derecruitment and reduced PaO_2 _and SaO_2_. While low V_T _strategies have been embraced, recent work indicates that survival from ARDS may be complicated by neurocognitive decline correlated to hypoxaemic periods during mechanical ventilation [23,24]. Richard et al. [25] advocate recruitment manoeuvres or increasing PEEP as alternative strategies to counteract low V_T _derecruitment. But recruitment manoeuvres did not show a sustained benefit for gas exchange in the ARDSNet trial [26] and increasing PEEP alone may be problematic. We have recently demonstrated that BVV is superior to an established recruitment manoeuvre to improve oxygenation in this animal model [8]. Martynowicz et al. [27], using parenchymal markers in an oleic acid injury model have demonstrated that PEEP restores airspace volume only at pressures that result in a universal increase in parenchymal stress. Eisner et al. [28] showed that the risk of barotrauma increased 1.67 fold for each 5-cm increment in PEEP and Esteban et al. [15] determined that increasing PEEP was an independent factor associated with mortality during mechanical ventilation.

A recent multi-centre trial has demonstrated no difference in patient outcome for low (8.3 cm H_2_O) versus high (13.2 cm H_2_O) levels of PEEP [29,30]. Since PEEP greater than 8 cm H_2O _is not beneficial to outcome, application of a variable signal to the ventilatory pattern with BVV may provide an alternative approach, producing net recruitment of previously fluid filled or atelectatic units without the potentially harmful effects of either increasing PEEP or the use of prolonged recruitment manoeuvres that deliver high levels of distending stress. Additionally, variable ventilation promotes release of endogenous surfactant, offering another mechanism for improvement in alveolar stability [14].

Potential criticisms of the present study include the use of PEEP and the use of a static P-V curve analysis for a dynamic application. Eight cm H_2_O PEEP was applied to both groups and resulted in peak airway pressures that exceeded the point of maximal compliance change for greater than 90% of breaths even in the Control group. The major consequence of this degree of PEEP is an upward shift of the P-V curve derived at zero end expiratory pressure (ZEEP) such that total volume would be greater at any point below *c*. However, in the models proposed by Hickling, this level of PEEP is associated with maintained convexity at lower V_T _and as such, has no effect on the interpretation of the results with regards to Jensen's inequality. We chose this level of PEEP to more closely approximate clinical practice, to provide evidence of a consistent lesion between animals with oleic acid administration and to ensure acceptable levels of gas exchange with the lower V_T _strategy. Initial attempts to analyse the P-V relationship on PEEP gave unreliable values for the point *P *= *c *- 1.317*d *in our hands; a consequence of inadequate definition of the lower asymptote in these earlier experiments.

We recognize that application of V_T _values obtained under "quasi" static conditions may not be directly applicable to the dynamic breathing cycle due to a shift to the right that occurs with increasing inspiratory flows and the increase in volume that may occur at the beginning of the dynamic inspirations due to recruitment from tidal volume independent of PEEP [31]. As such, our Experimental V_T _settings should be considered a first attempt to utilise information obtained from P-V curves to individualise BVV settings and may have also contributed to the lack of difference seen. However, a rightward shift of the curve implies that Jensen's inequality may apply over an even broader V_T _range provided that a convex P-V relationship is maintained. Examination of dynamic P-V loops from Rimensberger et al. [32] indicate that convexity persists. In addition, further analysis of the Venegas equation reveals its derivative closely resembles a Gaussian distribution [3]. Thus the probability density function of this equation indicates the likelihood of alveolar recruitment at a given airway pressure and the equation itself could be deemed the cumulative distribution function for alveolar recruitment. Airway opening leading to alveolar recruitment is curvilinear [13]. Under such circumstances Jensen's inequality implies that a noisy mean driving pressure could augment recruitment.

In an oleic acid lung injury model, Wilson and colleagues [33] suggested that oedematous lung did not open and close, but that alveoli changed from fluid-filled units to air-filled units with the P-V curve strongly sigmoidal. Following lung injury, at 3-5 cm H_2_O PEEP, convex curvature was seen in P-V curves over the range of inflation pressures seen in our study. They examined P-V curves for regional lung volumes of 1-2 mL, using the parenchymal marker technique. Thus in a similar model to ours, convexity was demonstrated for small regional lung volumes, suggesting applicability of Jensen's inequality with an oleic acid lung injury model of ARDS in the presence of PEEP. These authors further developed a mathematical model based on their findings. Until airway pressure exceeded 8 cm H_2_O (the level of PEEP in our study), the duct to the fluid-filled alveolus was assumed blocked by a liquid bridge. Above this pressure, the P-V curve was convex as the air bubble penetrated the mouth of the alveolus for various degrees of fluid filling. When the alveolus remained fluid-filled, the lung compliance was low. An abrupt change occurred as the air bubble entered the alveolus. At this transition, compliance rapidly increased with no change in alveolar tissue volume. Based on this modeling, when the lung is oedematous, noise added to the mean airway pressure signal will increase the likelihood of inducing the abrupt change in compliance, seen with entry of air bubbles into alveoli, a situation that would result in improved gas exchange.

The lack of difference between groups suggests that determination of a "mathematically optimal" V_T _may be clinically irrelevant during BVV, provided that ventilation is occurring on the convex portion of the P-V curve. Adhering to the ARDSNet algorithm in this study assured that ventilation occurred on the convex portion of the curve in all animals. Knowing this may be advantageous due to the difficulties applying static curves to dynamic conditions listed above. Moreover, rigorous definition of the P-V relationship requires a finite time off the ventilator, can be difficult to analyse clinically, and imposes a potential risk for instability in gas exchange and mechanics during the manoeuvre. PaO_2 _and compliance decreased in 18 of 22 animals immediately following P-V curve determination in the present study, providing credence to the above concerns. However, the results of the present study suggest that when ventilating with BVV, knowledge of the point of maximal compliance change on the convex portion of the P-V curve relative to V_T _determined using the ARDSNet algorithm might permit adjustments in V_T _in selected patients without the risk of excessive airway pressures. Finally, Jensen's inequality can be generalised and as such defining a simpler equation for the convex interval of the P-V curve under low V_T _conditions is possible.

We chose to examine only intratracheal IL-8 levels as a marker of inflammatory changes in the present study as previous work in our laboratory did not demonstrate measurable effects on tumour necrosis factor α, IL-6 or IL-10 in this porcine oleic acid model [11]. The high level of tracheal fluid IL-8 measured in both groups is comparable to our previous results. Similar cytokine levels for the two ventilatory approaches suggest that the inflammatory injury was comparable.

## Conclusion

In this porcine model of acute lung injury, "mathematically optimised" P-V curve fitting to calculate the mean V_T_/kg about which to centre variable ventilation yielded a broad range for this calculated volume. Although not clearly advantageous over the standard approach – low V_T _as determined by the ARDSNet algorithm – V_T _selected mathematically according to the point of maximal compliance change on the P-V curve, in combination with a variable pattern of ventilation, may permit some leeway in V_T _settings provided that airway pressures are maintained within acceptable limits. This study also indicates that the standard ARDSNet algorithm assures ventilation is occurring on the convex portion of the P-V curve with this model. Application of Jensen's inequality provides theoretical proof of why a noisy or variable ventilatory approach is advantageous under these circumstances.

## Competing interests

Dr. Mutch is co-founder of Biovar Life Support Inc., which has developed the mechanical ventilator described in this paper. Worldwide exclusive rights to this ventilator have been licensed to Respironics Inc. To date no ventilators have been sold clinically. In the event of sales of this ventilator, Dr. Mutch and the University of Manitoba would stand to gain financially. None of the other authors have a financial interest in the ventilator.

## Authors' contributions

M.R.G. supervised conduct of the experiments, helped analyse the data and helped write the paper. C.J.H. was responsible for conduct of the experiments as a fellow in the Anesthesia Laboratory. J.F.B. did the Jensen's inequality modelling and the statistical analysis related to the P-V curve fitting. L.G.G. helped with the experiments, data retrieval and collation and table and figure production. B.M.M. supervised the cytokine assays and their interpretation and helped write the paper. W.A.C.M. conceived the study, analysed and interpreted data and helped write the paper.
